# Effects of Intravenous Immunoglobulin and Acyclovir in Preventing Neonatal Varicella

**DOI:** 10.1155/2020/2709389

**Published:** 2020-06-18

**Authors:** Waritsara Piyanonpong

**Affiliations:** Department of Family Medicine, Trang Hospital, 69 Khokkhan Rd, Tubtiang, Muang, Trang 92000, Thailand

## Abstract

Neonatal varicella mostly results from maternal varicella. The disease can cause presentation ranging from mild symptoms to varicella pneumonia, hepatitis, meningoencephalitis, or fatality. If the mother develops symptoms implying varicella 4–5 days antepartum to 2 days postpartum, the mortality rate of the baby may reach 20%. We report a case of neonatal varicella from maternal varicella. The patient's mother initially developed maculopapular rash over her trunk 1 day after giving birth; she had a family member in the same household diagnosed with herpes zoster recently, and another member with diagnosed varicella, whose rash disappeared before the patient's birth. On the baby's third day of life, discrete vesicular rashes on erythematous background and discrete erythematous maculopapular rashes were found over his trunk, arms, and legs. The baby was subsequently diagnosed with neonatal varicella and was treated by intravenous immunoglobulin (IVIG) because there was no varicella zoster immunoglobulin (VZIG) available in the hospital, and also, intravenous acyclovir was given for 7 days. The rash completely resolved by the baby's fifth day of life, without any complications. The combination of IVIG and acyclovir might not effectively prevent neonatal varicella, but the medication could prevent the baby from developing serious complications and shorten the clinical course.

## 1. Introduction

Neonatal varicella is a rare condition, with the incidence of 2–6 per 100,000 live births per year [1]. Mostly, neonatal varicella is caused from maternal varicella, and the presentation of the disease is variable, ranging from mild symptoms to varicella pneumonia [2, 3], hepatitis [2, 4], or meningoencephalitis in disseminated disease [5, 6]. The worst outcome could be fatal [2, 3]. As maternal viremia happens, the transplacental infection occurs, which may start up to 48 hours before maternal rash develops. In neonates born to susceptible mothers who develop varicella symptoms from 4 to 5 days antepartum to 2 days postpartum, the mortality rate of the baby may reach 20–30% [1–3, 5]. Therefore, newborns whose mothers develop varicella 5 days prior to 2 days after birth should receive varicella zoster immunoglobulin (VZIG) in no time. Intravenous immunoglobulin (IVIG) can be used instead of VZIG if it is not available, which can be seen in many hospitals in Thailand. Moreover, the infant should be treated with intravenous acyclovir when lesions develop [2]. However, evidence supporting the use of acyclovir in neonatal varicella is limited to case reports and case series [7]. This case we are presenting is expected to be a supporting evidence of neonatal varicella treatment with IVIG and acyclovir.

## 2. Case Presentation

A term male baby was born to a 27-year-old Thai female by caesarean section due to prolonged second stage of labor at 39 + 3 weeks in Trang Hospital, Thailand. All physical examinations of the baby were within normal limits. The baby's vital signs were as follows: respiratory rate 58 breaths/min, blood pressure 63/33 mmHg, heart rate 140 beats per minute, and temperature 36.8°C. One day after the delivery, the mother developed maculopapular rash over her trunk, suggestive of chicken pox. The rash was discovered two days after the delivery, and the decision was made to move the baby to the isolation room of newborn nursery ward. There was no rash on the baby's skin on the transfer date.

There were 6 family members living together: the baby's mother, father, two other siblings, grandmother, and aunt. The baby's grandmother had herpes zoster for an unknown onset. Few weeks prior to the baby's birth, his aunt developed low-graded fever with vesicular skin eruptions, without seeking any medical care. In the family members' opinion, they thought it was likely to be varicella. The skin rash disappeared prior to the baby's birth. This history was not given on admission.

For treatment, IVIG 400 mg/kg/day was infused intravenously, started on the second day of life, the day when the baby was transferred to the isolation room. Other supportive treatments were also given. When the IVIG infusion was finished, intravenous acyclovir 25 mg/kg/day divided for every 8 hours was planned for seven days, started on the baby's second day of life. Expressed breast milk was collected in containers to restrict contact with mother.

**Figure 1:**
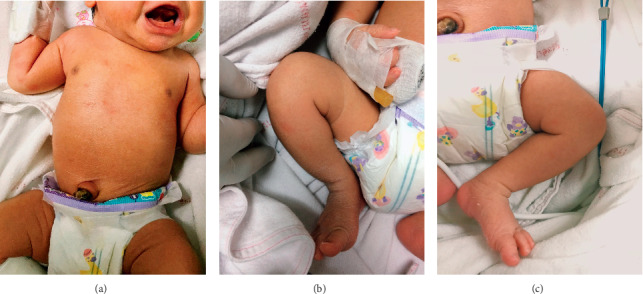
Neonatal varicella: the skin rash found appearing on the baby's third day of life.

A day later, on the baby's third day of life, discrete vesicular rashes on erythematous background with discrete erythematous maculopapular rashes were found over the baby's trunk, arms, and legs, as shown in Figures 1(a)–1(c). The baby was clinically diagnosed with neonatal varicella. The rash completely disappeared on the baby's fifth day of life. The baby was discharged on his ninth day of life, when acyclovir infusion of seven days was completed. During hospital admission, he was afebrile and doing well. The follow-up appointment with a pediatrician was made at 2 weeks after hospital discharge. On the follow-up visit, the baby was healthy, well-fed, and did not show any signs of complications.

## 3. Discussion

Neonatal varicella is not a commonly encountered disease, yet it is an important disease we should be able to detect early because it can be fatal in newborns [2, 3]. Varicella-zoster virus is the causative agent of this disease. It can be spread to another person by direct contact or through inhalation of aerosols from vesicular fluid of skin lesions, either from acute varicella or from zoster. The infection can also spread from aerosolized respiratory secretions [8]. The diagnosis of neonatal varicella is made clinically. In this case, the infection was caused by infected family members who were always in close contact with the patient's mother. Nobody in the family knew that this virus could be transmitted transplacentally and result in neonatal varicella.

Truthfully, if the information of the infected family was given at first, the mother would have been admitted to the isolation room because the risk of developing varicella during hospital admission was very high [9]. IVIG was given to the baby due to unavailability of VZIG in the hospital's medication list, which was also not available at some other hospitals in Thailand. From a previous study by Huang et al. [10], the combination of IVIG and intravenous acyclovir was administered to newborn infants whose mothers had developed varicella rash within 7 days before and 5 days after birth, and none of them developed varicella. In this case, the baby developed rash despite getting IVIG and acyclovir. After the baby was intravenously given IVIG and acyclovir, no complications occurred. The baby recovered fully and was discharged with no problems. In conclusion, the combination of IVIG and acyclovir might not effectively prevent neonatal varicella in this case, but the medication could prevent the baby from serious complications and shorten the clinical course.

## Abbreviations

IVIG: Intravenous immunoglobulin
kg: Kilogram
mg: Milligram
VZIG: Varicella zoster immunoglobulin.
